# Mechanical ventilation settings during weaning from venovenous extracorporeal membrane oxygenation

**DOI:** 10.1186/s13613-024-01359-2

**Published:** 2024-09-04

**Authors:** Maria Teresa Passarelli, Matthieu Petit, Roberta Garberi, Guillaume Lebreton, Charles Edouard Luyt, Marc Pineton De Chambrun, Juliette Chommeloux, Guillaume Hékimian, Emanuele Rezoagli, Giuseppe Foti, Alain Combes, Marco Giani, Matthieu Schmidt

**Affiliations:** 1 Department of Medicine and Surgery, University of Milano-Bicocca, Monza, Italy; 2 Department of Emergency and Intensive Care, Fondazione IRCCS San Gerardo Dei Tintori, Monza, Italy; 3 Assistance Publique-Hôpitaux de Paris, Service de Médecine Intensive-Réanimation, Institut de Cardiologie, Hôpital Pitié-Salpêtrière, 47 Boulevard de L’Hôpital, 75651 Paris Cedex 13, France; 4 Sorbonne Université, INSERM, UMRS_1166-ICAN, Institute of Cardiometabolism and Nutrition, Paris, France; 5 Assistance Publique-Hôpitaux de Paris, Thoracic and Cardiovascular Department, Pitié-Salpêtrière Hospital, 75651 Paris Cedex 13, France

**Keywords:** Acute respiratory distress syndrome, Extracorporeal membrane oxygenation, Weaning, Mechanical ventilation, Spontaneous breathing

## Abstract

**Background:**

The optimal timing of weaning from venovenous extracorporeal membrane oxygenation (VV ECMO) and its modalities have been rarely studied.

**Methods:**

Retrospective, multicenter cohort study over 7 years in two tertiary ICUs, high-volume ECMO centers in France and Italy. Patients with ARDS on ECMO and successfully weaned from VV ECMO were classified based on their mechanical ventilation modality during the sweep gas-off trial (SGOT) with either controlled mechanical ventilation or spontaneous breathing (i.e. pressure support ventilation). The primary endpoint was the time to successful weaning from mechanical ventilation within 90 days post-ECMO weaning.

**Results:**

292 adult patients with severe ARDS were weaned from controlled ventilation, and 101 were on spontaneous breathing during SGOT. The 90-day probability of successful weaning from mechanical ventilation was not significantly different between the two groups (sHR [95% CI], 1.23 [0.84–1.82]). ECMO-related complications were not statistically different between patients receiving these two mechanical ventilation strategies. After adjusting for covariates, older age, higher pre-ECMO sequential organ failure assessment score, pneumothorax, ventilator-associated pneumonia, and renal replacement therapy, but not mechanical ventilation modalities during SGOT, were independently associated with a lower probability of successful weaning from mechanical ventilation after ECMO weaning.

**Conclusions:**

Time to successful weaning from mechanical ventilation within 90 days post-ECMO was not associated with the mechanical ventilation strategy used during SGOT. Further research is needed to assess the optimal ventilation strategy during weaning off VV ECMO and its impact on short- and long-term outcomes.

**Supplementary Information:**

The online version contains supplementary material available at 10.1186/s13613-024-01359-2.

## Background

The rationale for venovenous extracorporeal membrane oxygenation (V-V ECMO) utilization is to ensure adequate gas exchange while allowing ventilator settings that enhance ventilator-induced lung injury (VILI) prevention, a key contributor to morbidity and mortality in acute respiratory distress syndrome (ARDS) [1]. The evidence supporting the use of ECMO is becoming more robust, as demonstrated in multicenter clinical trials [2, 3], bayesian analysis [4], and meta-analyses [5–7]. On these bases, the latest European guidelines on ARDS recommend V-V ECMO utilization in high-volume centers [8]. However, there are still pending research questions in that field, especially on the timing and the modalities of ECMO weaning. Once the lung function has sufficiently recovered [2, 9], the sweep gas flow is turned off, and the patient’s oxygenation and decarboxylation are both closely monitored for 6–24 h to assess the ability to be decannulated and liberated from V-V ECMO [9]. However, the optimal timing of this weaning trial and its modalities have not been well standardized and are mainly based on clinician preferences and expert opinion [10–13]. Indeed, two specific periods for ECMO weaning could be identified. Early weaning from V-V ECMO, when the patient is still on controlled ventilation with deep sedation, could reduce the risks of ECMO-related complications and costs. However, this approach may jeopardize the prevention of VILI and expose the patients to the need for a new ECMO run [13, 14]. On the other hand, waiting for an awake patient capable of breathing spontaneously on V-V ECMO may necessitate time [15]. This strategy could theoretically prolong the duration of ECMO, thereby increasing the patient's vulnerability to ECMO-related complications. On the other hand, it may also be associated with multiple physiological benefits, such as improved ventilation-perfusion matching, preserved respiratory muscle function, and decreased need for sedatives [16]. If this can be achieved while maintaining adequate control of the respiratory drive, it may favor lung healing and thereby facilitate liberation from ECMO. To date, the impact of different ventilation strategies during ECMO weaning on outcomes has been poorly investigated, although some authors have recently outlined ventilatory and clinical parameters that can predict unfavorable outcomes [14, 17]. Furthermore, the decision to discontinue ECMO or mechanical ventilation first is still a matter of debate [18, 19]**.**

The objectives of this multicenter, retrospective study were (1) to describe the mechanical ventilation settings used at the time of V-V ECMO weaning in two experienced ECMO centers; (2) to compare two different approaches during the weaning process, namely controlled mechanical ventilation versus spontaneous assisted breathing, in terms of mechanical ventilation duration, ICU and hospital lengths of stay, and mortality after ECMO weaning.

## Methods

### Study design and patients

This study retrospectively included patients with severe ARDS (according to the Berlin Definition [20]) treated with ECMO in two university tertiary medical centers between January 2015 and December 2022. The medical ICUs from Pitié-Salpêtrière Hospital, Paris, and IRCCS San Gerardo dei Tintori Hospital, Monza, are among the largest and the most experienced ECMO centers in France and Italy, respectively. All consecutive patients with ARDS who received V-V ECMO or other ECMO settings (i.e., veno-arterial, V-A, or veno-arteriovenous, V-AV) during the study period were screened. Only patients weaned alive from V-V ECMO were included in this study. The exclusion criteria were no intubation on ECMO, bridge to lung transplant, or extubated before ECMO weaning.

Following ethical standards of local Institutional Review, no informed consent was required for this retrospective, observational study. The National Commission for Informatics and Liberties (no. 2217028v0) and the Comitato Etico Brianza (ref. NP3369) approved the data collection for this study.

### Management of sedation and mechanical ventilation during ECMO

Deep intravenous (propofol or midazolam) or volatile (isoflurane [21]) sedation was maintained in the early phase of the disease (i.e. Richmond Agitation-Sedation Scale (RASS) [22] ≤ − 3), with the addition of neuromuscular blockade in case of patient-ventilator asynchronies. Light sedation (− 2 ≤ RASS ≤ − 1) with either a low dose of propofol or dexmedetomidine was used when the clinical condition was improving. The ventilation strategies used generally followed the EOLIA protocol [2]. Ultraprotective ventilation was provided either by volume or pressure-control modality, with a tidal volume of 6 ml/kg or below, adjusted to maintain a driving pressure below 15 cmH_2_O. Positive end-expiratory pressure was set at 10 cmH_2_O or more, and the respiratory rate was maintained between 10 and 20 cycles/min.

### Sweep gas-off trial (SGOT)

The ventilatory and blood gas parameters at the end of the sweep gas-off trial (SGOT) that preceded the liberation from ECMO were recorded. The SGOT trial consisted of turning off the sweep gas flow while maintaining ECMO blood flow > 3 L/min, to avoid clotting [9]. The test duration ranges from 6 to 12 h (i.e. Monza, Italy) to 24 h (i.e. Paris, France) and aims to emulate gas exchanges with mechanical ventilation only. Based on lung function improvement, respiratory mechanics, and gas exchanges [9], the clinician in charge of the patient decided to perform an SGOT on controlled mechanical ventilation or spontaneous breathing with pressure support. Based on these mechanical ventilation modalities at the time of SGOT, patients were classified as *Controlled Ventilation Group* (i.e. patients who underwent the trial either on Volume-Control Ventilation, Pressure-Control Ventilation, Airway Pressure Release Ventilation, or Pressure-Control Bi-Level Positive Airway Pressure) or *Spontaneous Breathing Group* (i.e. patients on pressure support ventilation during the SGOT). Successful weaning criteria, including lung mechanics and gas exchanges, according to mechanical ventilation modalities during SGOT have been described elsewhere [2, 23] and are reported in Additional file 1.

### Data collection

We collected data before ECMO implantation and at the time of ECMO weaning. Pre-ECMO ventilation settings and blood gas, Sepsis-related Organ Failure Assessment (SOFA) score, need for renal replacement therapy (RRT), cause of ARDS (i.e., viral pneumonia, bacterial pneumonia, autoimmune cause, or others), adjunct therapies before ECMO start, and ECMO management were also reported. The ventilation settings and the blood gas parameters were collected right before VV ECMO implantation. The SOFA score was intended as the one at the admission to the ECMO unit.

### Outcome variables

The primary outcome was the time to successful weaning from mechanical ventilation within 90 days following ECMO discontinuation. Successful weaning from mechanical ventilation was defined as the removal of the endotracheal tube or tracheostomy cannula (for tracheostomized patients) without the need for reintubation in the following 72 h. Death or new ECMO run within 90 days after ECMO weaning were considered as competing events. Secondary outcomes were ventilator-associated pneumonia (VAP), ICU and hospital length of stay and mortality. VAP was diagnosed in patients who were on mechanical ventilation for at least 48 h and showed significant quantitative growth (≥ 10^4^ colony-forming units (CFU)/mL) of at least one pathogen in the Broncho-Alveolar Lavage fluid sample [24, 25].

### Statistical analyses

This study followed the STROBE (Strengthening the Reporting of Observational Studies in Epidemiology) recommendations for reporting cohort studies [26]. No power calculation or sample size computation was performed.

Baseline characteristics are reported as proportions (%) for categorical variables and as median [interquartile range, IQR] for continuous variables. The primary endpoint was the time to successful mechanical ventilation weaning within the 90 days following ECMO weaning, in the presence of the competing risks of death and second ECMO run according to the two mechanical ventilation groups. Day 0 of mechanical ventilation was considered as the date of ECMO weaning. The cumulative incidence curves for these competing events were drawn for each group. The cumulative incidence of successful mechanical ventilation weaning was compared between groups using a Gray test. The subdistribution hazard ratios (sHR) were estimated (with their 95% confidence interval, CI) for the competing events using a Fine and Gray competing risk regression. Baseline variables (i.e., obtained at the time of ECMO start) and ECMO weaning variables (i.e., obtained at the time of ECMO weaning) included in the multivariable model were defined a priori* based* on the available literature. Baseline variables included age, body mass index, COVID-19-related ARDS (yes/no), pre-ECMO PaO_2_/FiO_2_, and pre-ECMO SOFA. ECMO weaning variables were ventilation mode and compliance of the respiratory system at the time of the SGOT, ECMO duration, prone positioning on ECMO, VAP, pneumothorax, and renal replacement therapy before the SGOT. No imputation for missing data was performed. Log linearity was graphically assessed for the quantitative variable’s effects using restricted cubic splines. Additionally, a sensitivity analysis was performed for the subgroup of patients with COVID-19.

Categorical outcomes were compared with chi-square or Fisher’s exact tests, and continuous outcomes with Student’s t-test or Wilcoxon’s test, as appropriate. All analyses were conducted at the two-sided α risk of 5%. All analyses were performed using R software (R Foundation for Statistical Computing, Vienna, Austria), version 4.1.3.

## Results

### Study population

**Fig. 1:**
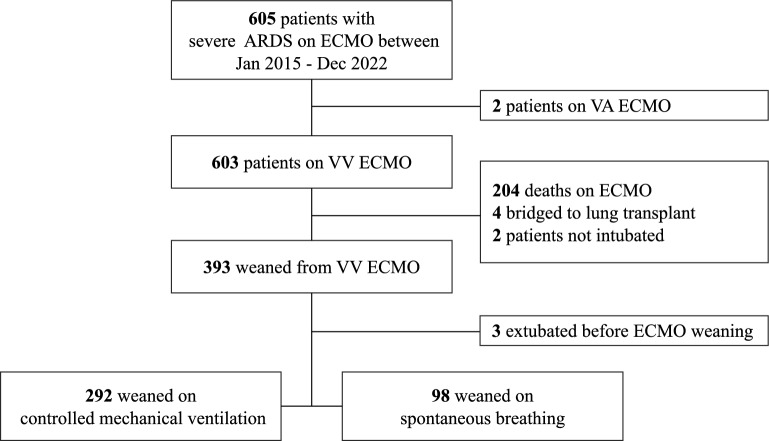
Study flowchart. *ARDS*, acute respiratory distress syndrome; *V-A*, *ECMO* veno-arterial extracorporeal membrane oxygenation; *V-V ECMO*, venovenous extracorporeal membrane oxygenation

The study flowchart is presented in Fig. 1. Of 603 patients receiving VV ECMO primarily for ARDS during the 7-year study period, 390 (median age 50 (IQR 41; 57) years) underwent a successful ECMO weaning and were included in our study. Two hundred and ninety-two (75%) patients had an SGOT on Controlled Ventilation whereas 98 (25%) were on Spontaneous Breathing. The baseline and pre-ECMO characteristics of these two study groups are reported in Table 1**.** Briefly, patients in the controlled ventilation group were significantly younger, had a higher body mass index, and were more frequently retrieved on ECMO to the two referral centers. The most frequent comorbidities were hypertension, diabetes, and chronic respiratory disease. Notably, 27 (7%) patients were immunocompromised at cannulation. The main cause of ARDS was COVID-19 in both groups, followed by bacterial pneumonia and viral non-COVID pneumonia. Patients in the controlled ventilation group had a significantly longer time between mechanical ventilation and ECMO than those in the spontaneous breathing group (3 [1–6] vs 1 [1–5] days, respectively, *p* = 0.004) and showed a lower Pre-ECMO PaO_2_/FiO_2_ ratio and a higher PaCO_2_. Pre-ECMO lung mechanics were more severe in the controlled ventilation group, with a significantly lower PEEP, a higher plateau pressure and respiratory rate, and a lower tidal volume. In this subgroup, before ECMO implementation, nitric oxide, and prone positioning were used more frequently. On the other hand, neuromuscular blockades were used similarly in both groups (Table 1).

**Table 1:** Baseline characteristics according to the ventilation modalities during sweep gas-off trial

	All patients	Controlled ventilation	Spontaneous breathing	*p*-value
	*N* = 390	*N* = 292	*N* = 98	
ECMO center				<0.001
Paris	285 (73)	278 (95)	7 (7)	
Monza	105 (27)	14 (5)	91 (93)	
Female sex	136 (35)	97 (33)	39 (40)	0.289
Age, year	50 [41; 57]	49 [39; 56]	52 [44; 59]	0.009
BMI, kg/m^2^	31.2 [27.5; 37.5]	31.7 [27.8; 38.5]	30.5 [26.2; 35.2]	0.038
Pregnancy	16 (4)	14 (5)	2 (2)	0.269
Comorbidities				
Hypertension	144 (37)	111 (38)	33 (34)	0.401
Diabetes	93 (24)	79 (27)	14 (14)	0.011
Chronic respiratory disease^a^	69 (18)	60 (20)	9 (9)	0.012
Chronic heart failure	31 (8)	24 (8)	7 (7)	0.900
Chronic renal disease	15 (4)	13 (4)	2 (2)	0.392
Immunocompromised status	27 (7)	15 (5)	12 (12)	0.030
ARDS etiology				0.091
COVID-19	184 (47)	151 (52)	33 (34)	
Bacterial	97 (25)	66 (23)	31 (32)	
Viral Other	55 (14)	34 (12)	21 (21)	
Autoimmune	14 (4)	7 (2)	7 (7)	
Other	12 (3)	9 (3)	3 (3)	
Unknown	18 (5)	15 (5)	3 (3)	
MV-to-ECMO interval (days)	3 [1; 6]	3 [1; 6]	1 [1; 5]	0.004
Retrieved on ECMO	310 (79)	253 (87)	57 (58)	<0.001
Pre-ECMO blood gas				
PaO_2_/FiO_2,_ mmHg	65 [54; 75]	61 [51; 70]	71 [57; 88]	<0.001
PaCO_2_, mmHg	56 [49; 65]	57 [49; 67]	54 [47; 62]	0.026
pH	7.33 [7.24; 7.39]	7.32 [7.22; 7.39]	7.35 [7.28; 7.39]	0.097
Pre-ECMO ventilation				
PEEP, cmH_2_O	12 [10; 15]	12 [10; 14]	14 [12; 15]	0.005
Plateau pressure, cmH_2_O	30 [28; 32]	30 [30; 32]	28 [25; 30]	<0.001
Tidal volume, ml/kg ibw	5.9 [4.5; 6.5]	5.8 [2.8; 6.2]	6.4 [5.6; 7.3]	<0.001
Respiratory rate, cycles/min	28 [25; 30]	30 [26; 32]	26 [22; 29]	<0.001
Pre-ECMO SOFA score	11 [8; 13]	12 [8; 14]	8 [5; 11]	<0.001
Pre-ECMO adjunct therapies				
NMBA	382 (98)	286 (98)	96 (98)	1.000
Prone Positioning	293 (75)	232 (79)	61 (62)	0.001
iNO	156 (40)	128 (44)	28 (29)	0.017
High dose corticosteroids	47 (12)	32 (11)	15 (15)	0.335
Pre-ECMO RRT	32 (8)	23 (8)	9 (9)	0.845
MV on ECMO	393 (100)	292 (100)	98 (100)	
ECMO configuration				<0.001
VV Fem-Jug	289 (74)	266 (91)	23 (23)	
VV Fem-Fem	92 (24)	18 (6)	74 (75)	
Other	9 (2)	8 (3)	1(1)	

Values are expressed as median (interquartile range) or *n* (%)

^a^Chronic respiratory disease includes asthma, chronic obstructive pulmonary disease, or restrictive lung disease

BMI = body mass index, MV = mechanical ventilation, iNO = inhaled nitric oxide, NMBA = neuromuscular blockades, RRT = renal replacement therapy

### Characteristics during the SGOT

The characteristics and lung mechanics at the time of SGOT are presented in Table 2. At the time of SGOT, patients were on ECMO for 13 [7–29] and 12 [9–18] days (*p* = 0.398) in the controlled ventilation and the spontaneous breathing groups, respectively. When compared to patients in the spontaneous breathing group, patients in the controlled ventilation group had a significantly lower tidal volume, PEEP, and higher respiratory rate. Similarly, lower static respiratory system compliance and higher plateau pressure and driving pressure were reported in the controlled ventilation group.

**Table 2:** Characteristics during sweep gas-off trial according to the ventilation modalities

	All patients (*N* = 390)	Controlled ventilation (*N* = 292)	Spontaneous breathing (*N* = 98)	*p*-value
ECMO duration, days	13 [7; 27]	13 [7; 29]	12 [9; 18]	0.398
Ventilation during SGOT				<0.001
Controlled ventilation				
VCV	237 (60)	237 (81)	0 (0)	
PC-APRV	40 (10)	40 (14)	0 (0)	
PC-BiPAP	13 (3)	13 (4)	0 (0)	
PCV	2 (0.5)	2 (1)	0 (0)	
Spontaneous Breathing				
PSV	26 (7)	0 (0)	26 (27)	
PSV + sigh	72 (18)	0 (0)	72 (73)	
Ventilatory parameters				
Tidal Volume, ml/kg ibw	6.1 [5.7; 7.1]	6 [5.6; 6.4]	7.6 [6.7; 9]	<0.001
Respiratory rate, cycles/min	26 [20; 29]	28 [25; 30]	15 [14; 19]	<0.001
PEEP, cmH_2_O	12 [8; 14]	10 [8; 14]	12 [10; 14]	<0.001
Plateau Pressure, cmH_2_O	26 [23; 28]	27 [24; 29]	22 [21; 25]	<0.001
Driving Pressure, cmH_2_O	14 [11; 18]	15 [13; 19]	11 [9; 13]	<0.001
Compliance, ml/cmH_2_O	29 [21; 40]	25 [19; 33]	46 [36; 59]	<0.001
Pressure Support level, cmH_2_O	10 [8; 10]	–	10 [8; 10]	
FiO_2_, %	40 [40; 50]	40 [40; 50]	40 [40; 50]	0.161
Blood gas parameters				
PaO_2_, mmHg	89 [79; 110]	88 [77; 108]	96 [83; 115]	0.003
PaCO_2_, mmHg	43 [38; 48]	41 [38; 46]	47 [43; 51]	<0.001
pH	7.43 [7.39; 7.46]	7.43 [7.38; 7.47]	7.43 [7.40; 7.46]	0.812
Lactate, mmol/L	1 [0.7; 1.3]	1 [0.7; 1.3]	1 [0.8; 1.4]	0.210
Bicarbonates, mmol/L	28.6 [24.7; 31.6]	28.0 [23.9; 31.0]	30.5 [28.0; 33.2]	<0.001

Values are expressed as median (interquartile range) or *n* (%)

*VCV*, volume control ventilation; *PC-APRV*, pressure control-airway pressure release ventilation; *PC-BiPAP*, pressure control-bilevel positive airway pressure; *PCV*, pressure control ventilation; *PSV*, pressure support ventilation; *PEEP*, Positive End Expiratory Pressure; *PaO2*, partial pressure of arterial oxygen; *PaCO2*, *p*artial pressure of arterial carbon dioxide; *RRT*, renal replacement therapy; *VAP*, ventilator-associated pneumonia

### Primary and secondary outcomes

**Fig. 2:**
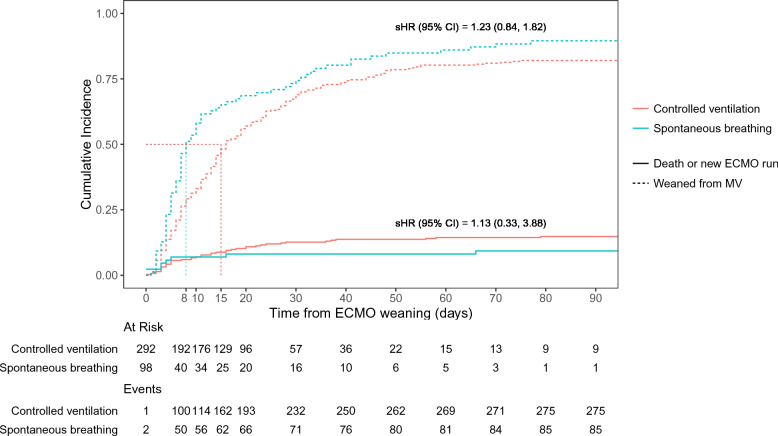
Cumulative incidence function for the events of mechanical ventilation successful weaning and death or second run of ECMO, according to mechanical ventilation modalities during sweep gas-off trial. *sHR*, subdistribution hazard ratio; *MV*, mechanical ventilation

The probability of successful weaning from mechanical ventilation within 90 days of ECMO discontinuation was not significantly different between the two groups (sHR, 1.23 [95% CI 0.84–1.82], *p* = 0.301) (Fig. 2). Death or a second ECMO run, the competing component of the primary outcome, was not significantly different between groups (sHR, 1.13 [95% CI [0.33–3.88]), *p* = 0.802) (Fig. 2). Patients in the spontaneous breathing group had a lower unadjusted length of stay in the ICU and the hospital and lower hospital mortality after ECMO weaning. ECMO-related complications, such as severe bleeding or ischemic stroke were not different between the two groups. Ventilator-associated pneumonia was more frequently recorded in the controlled ventilation group (Table 3).

**Table 3:** ECMO management, complications, and outcomes according to the mechanical ventilation modalities during sweep gas-off trial

	All patients *N* = 390	Controlled ventilation *N = *292	Spontaneous breathing *N = *98	*P *overall
Prone positioning on ECMO	244 (62)	187 (64)	57 (56)	0.215
Tracheostomy	163 (42)	124 (42)	39 (40)	0.730
RRT	169 (43)	114 (39)	55 (54)	0.010
At least one VAP	268 (68)	210 (72)	58 (57)	0.010
Any severe bleeding	89 (23)	69 (24)	20 (20)	0.604
Hemothorax	9 (2)	6 (2)	3 (3)	0.702
Gastrointestinal bleeding	25 (6)	22 (8)	3 (3)	0.183
Other bleeding	55 (14)	44 (15)	11 (11)	0.430
Ischemic stroke	5 (1)	5 (2)	0 (0)	0.344
Pneumothorax	47 (12)	34 (12)	13 (13)	0.805
MV duration post ECMO weaning, d	13 [6;28]	14 [7;28]	8 [4;19]	0.002
Total ICU LOS, d	44 [24;64]	48 [28;67]	26 [21;49]	<0.001
ICU LOS post-ECMO weaning, d	16 [9;31]	19 [11;34]	10 [7;21]	<0.001
Total hospital LOS, d	68 [41;92]	72 [46;95]	48 [35;73]	<0.001
Hospital LOS post-ECMO weaning, d	35 [23;57]	39 [24;62]	28 [18;48]	0.001
ICU mortality post ECMO weaning	36 (9)	32 (11)	4 (4)	0.091
Hospital mortality post ECMO weaning	42 (11)	38 (13)	4 (5)	0.041

Values are expressed as median (interquartile range) or *n* (%)

*d*, days; *MV*, mechanical ventilation; *ICU*, intensive care unit; *LOS*, length of stay

**Fig. 3:**
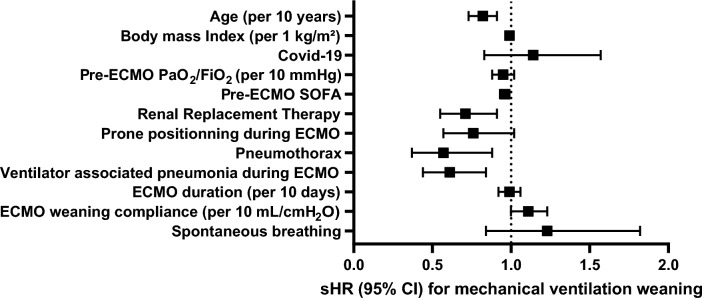
Association of covariates with the 90-day adjusted probability of successful weaning from mechanical ventilation after ECMO decannulation in the multivariable model, expressed using sHR (points) with their 95% CI (error bars). *sHR*, subdistribution hazard ratio*.* The model was performed on 355 patients due to missing data

After adjustment to the patient’s characteristics and events occurring during the ECMO run, being on spontaneous ventilation during SGOT was not associated with a greater probability of successful mechanical ventilation weaning. Conversely, older age, ventilator-associated pneumonia on ECMO, pneumothorax, and RRT in ICU were significantly associated with a lower probability of successful weaning from mechanical ventilation at 90 days. A shorter ECMO duration and greater static compliance during SGOT were associated with a significant increase in the probability of weaning from mechanical ventilation (Fig. 3). Moreover, similar risk factors for successful weaning from mechanical ventilation at 90 days were found when the analysis was performed for the subgroup of patients with COVID-19 (see Additional file 2).

## Discussion

This study investigated ventilatory modalities and subsequent outcomes in 390 patients weaned from V-V ECMO. The analysis revealed no significant difference in the rate of successful mechanical ventilation weaning after SGOT, accounting for death and the need for a second ECMO run as competing events. Despite similar ECMO duration, patients on spontaneous breathing during SGOT had a shorter ICU and hospital length of stay, when compared to patients on controlled mechanical ventilation. However, after adjusting for covariates, spontaneous breathing during SGOT was not independently associated with a higher probability of being weaned from mechanical ventilation, as compared to controlled ventilation. Noticeably, spontaneous breathing during ECMO weaning was not associated with a higher incidence of ECMO-related complications.

Strong evidence to guide mechanical ventilation settings during V-V ECMO is still lacking. Current recommendations rely on experts’ opinions and ventilatory settings used in the ECMO groups in two recent RCTs [2, 3, 9]. Ultra-protective lung ventilation settings could enhance VILI prevention on ECMO [27], as suggested in the LIFEGARDS study [28] and several reviews [23, 29, 30]. Nonetheless, the association between ventilatory parameters while on ECMO and outcomes has not been well established, with studies showing conflicting results [28, 31]. Literature regarding mechanical ventilation settings during ECMO weaning is even more scarce since this topic has received very little attention. Limited data offer guidance about when and how to perform an SGOT [10–12, 23, 32]. Al-Fares et al. demonstrated that patients exhibiting higher tidal volumes, heart rate, ventilatory ratio, and esophageal pressure swings during SGOT have a decreased likelihood of achieving a safe liberation from VV ECMO [14]. Similarly, Gerhardinger et al*.* recently identified higher respiratory rate and PaCO_2_ before SGOT as independent risk factors for ECMO weaning failure while, during the trial, impaired oxygenation was the most relevant risk factor of ECMO weaning failure [33]. A higher PaCO_2_ and respiratory rate at the time of ECMO decannulation were also associated with prolonged mechanical ventilation and ICU length of stay following decannulation, whereas high PEEP seemed protective [17]. Our multivariate analysis showed that pneumothorax, ventilation-associated pneumonia, and renal replacement therapy during ECMO, all surrogates of patient severity, were independently associated with a lower likelihood of being successfully weaned from mechanical ventilation at day 90.

The ECMO duration until SGOT was unexpectedly found to be not different between the two groups in our study. One could argue that waiting for patients to be able to undergo spontaneous breathing ventilation could expose them to a longer ECMO run and therefore greater likelihood of ECMO-related complications. Interestingly, severe bleeding and pneumothorax incidence were similar between the two groups. These findings are reassuring, suggesting that maintaining V-V ECMO support until being on spontaneous ventilation is not associated with worse outcomes compared to controlled mechanical ventilation. A weaning ECMO strategy that encourages spontaneous breathing before or during an SGOT may facilitate liberation from ECMO by the inherent physiological benefits of spontaneous breathing efforts, such as the recruitment of the dorsal-dependent lung regions. Moreover, as compared to using ultra-low tidal volumes (1–2 mL/kg of predicted body weight) alongside prolonged infusion of neuromuscular blockers, this approach might be associated with more favorable outcomes [34]. Additionally, it may reduce costs and resource consumption following decannulation.

Initially, we hypothesized that patients weaned from ECMO on spontaneous breathing would require a longer ECMO duration. However, our findings contradicted this hypothesis, as the ECMO durations in both study groups were similar. Nonetheless, the lower pre-ECMO severity in the spontaneous breathing group, which facilitated faster lung function improvement, might partially account for these results. Further investigation is still necessary to determine the optimal timing for SGOT. Noticeably, some patients may be "forced" to undergo the SGOT despite not fulfilling the respiratory mechanic prerequisites for ECMO weaning criteria, due to severe ECMO complications or a lack of clinical improvement after a prolonged ECMO course [35].

Although it is to date the largest study on mechanical ventilation modalities during SGOT, we acknowledge that our study has several limitations. First, given its retrospective design, it is not possible to establish direct causal relationships, but only associations. Furthermore, for the same reason, some potentially important data might be missing. For instance, we did not have access to the data regarding the timing and number of previous failed SGOTs before the one preceding the liberation from ECMO. The ventilatory modes used at those times were not collected. Similarly, the surrogates of the respiratory drive (e.g., P0.1, delta Pocc, and Pressure-Muscle-Index) at the time of SGOT, which were recorded in the spontaneous breathing group, have not been collected as well. Second, we included patients from two distinct ECMO centers where ECMO practices may slightly differ (e.g., ECMO cannulation sites, and mode of ventilation during ECMO weaning…). Unfortunately, a Fine and Gray model with a random effect on the center does not exist to date. Notably, spontaneous breathing at the SGOT was the preferred modality at San Gerardo Hospital, while controlled mechanical ventilation was mainly used at La Pitié-Salpêtrière Hospital. Third, we did not collect data regarding the use of adjunct therapies for ARDS post-ECMO weaning in both groups, which may also have an important impact on economic and human resources. Fourth, we did not gather information on the level of sedation, neuromuscular blockade use, and fluid balance during the ECMO course, which may have significantly impacted the study outcomes. Lastly, our follow-up was limited to 90 days after ECMO weaning. Exploring the impact of these ventilation strategies on long-term lung function or psychological status could be valuable.

## Conclusion

A strategy comprising spontaneous breathing during SGOT was not associated with a higher incidence of successful weaning from mechanical ventilation, compared to controlled mechanical ventilation. However, this approach appears to be safe and not associated with more ECMO-related complications. Further research is needed to assess the optimal ventilation strategy during weaning off V-V ECMO and its impact on short- and long-term outcomes.

## Supplementary Information

**Additional file 1.** Prerequisites for weaning trial, weaning trial protocol, and criteria for successful trial according to the two groups.

**Additional file 2.** Association of covariates with the 90-day adjusted probability of successful weaning from mechanical ventilation after ECMO decannulation in the multivariable model analyzing the subgroup of COVID-19 patients, expressed using sHR with their 95% CI.

## Data Availability

The datasets analyzed during the current study are available from the corresponding author upon reasonable request.

## Abbreviations

ARDS: Acute respiratory distress syndrome
COVID-19: Coronavirus disease 2019
ECMO: Extracorporeal membrane oxygenation
FiO2: Fraction of inspired oxygen
IBW: Ideal body weight
ICU: Intensive care unit
MV: Mechanical ventilation
PaO2: Partial pressure of alveolar oxygen
PBW: Predicted Body Weight
PEEP: Positive end-expiratory pressure
SAPS II: Simplified Acute Physiology Score II
SARS-CoV-2: Severe acute respiratory distress syndrome coronavirus 2
SOFA: Sequential Organ Failure Assessment
SpO2: Oxygen saturation measured by pulse oximetry
