# Supplementary material for: Mechanical ventilation settings during weaning from venovenous extracorporeal membrane oxygenation
Source: Ann Intensive Care. 2024 Sep 4;14:138. doi: 10.1186/s13613-024-01359-2 (PMC11374948; doi:10.1186/s13613-024-01359-2)
Supplement: Supplementary file 1 — Additional file 1. Prerequisites for weaning trial, weaning trial protocol, and criteria for successful trial according to the two groups. [file 13613_2024_1359_MOESM1_ESM.docx]

**Additional file 1: Prerequisites for weaning trial, weaning trial protocol, and criteria for successful trial according to the two groups**

|  | **Controlled Ventilation** | **Spontaneous Breathing** |
| --- | --- | --- |
| **Prerequisites for ECMO weaning trial** | - (Full) sedation +/- NMBA - TV 6mL/kg IBW; RR ≤ 28/min; PEEP 6-14 cmH_2_O; FiO_2_ ≤60%   *With resulting :*   - Pplat ≤ 28 cmH_2_O or - Pplat ≤ 34 cmH_2_O if BMI >34 kg/m2 | - RR ≤ 25/min; PEEP 6-14 cmH_2_O; FiO_2_ ≤50% - Compliance _RS_ >0.4 ml/cmH_2_O/kg of IBW   *With resulting :*   - Driving pressure ≤ 14cmH_2_O |
| **ECMO Weaning trial** | - Turn off the sweep gas flow - Maintain ECMO flow >3L/min   *If:*   - SpO2 ≥90% with FiO_2_ ≤60%   *Then perform :*   - Blood gas at H1, H3, and H6 | - Turn off the sweep gas flow - Maintain ECMO flow >3L/min   *If :*   - SpO_2_ ≥90% with FiO_2_ ≤50% - RR ≤ 25/min - Driving pressure <15cmH_2_O   *Then perform :*  Blood gas at H1, H3 |
| **Criteria for ECMO withdrawing)** | *Withdraw ECMO if after a 24-hour weaning trial:*   - PaO_2_ ≥ 60 mmHg, SaO_2_ ≥ 90%, with FiO_2_ ≤ 60% - PCO2 ≤ 50 mmHg or pH ≥7.36, with RR ≤ 28/min - Pplat ≤ 28 cmH_2_O *or* ≤ 34 cmH_2_O if BMI >34 kg/m^2^ - Driving pressure ≤ 14cmH2O - and if no signs of acute cor pulmonale | *Withdraw ECMO if after a 6-12-hour weaning trial:*   - pH ≥7.35, with RR ≤ 25/min - Compliance _RS_ >0.4 ml/cmH_2_O/kg of IBW - Driving pressure ≤ 14cmH_2_O |

*NMBA* neuromuscular blocking agents*, TV* tidal volume*, IBW* ideal body weight*, PEEP* positive end-expiratory pressure*, Pplat* plateau pressure*, BMI,* body mass index*, RR* respiratory rate*.*

**Additional file 2: Association of covariates with the 90-day adjusted probability of successful weaning from mechanical ventilation after ECMO decannulation in the multivariable model stratified on COVID-19, expressed using sHR with their 95% CI**

|  | **Subdistribution hazard ratio.**  **(95% CI)** | ***P* value** |
| --- | --- | --- |
| Age, per 10 years | 0.78 (0.69 – 0.88) | <0.001 |
| Body mass index, per 10 kg/m^2^ | 0.88 (0.74 – 1.04) | 0.140 |
| Pre-ECMO PaO_2_/FiO_2_, per 10mmHg | 0.94 (0.87 – 1.02) | 0.141 |
| Pre-ECMO SOFA | 0.96 (0.93 – 1.00) | 0.047 |
| Renal replacement therapy | 0.78 (0.59 – 1.03) | 0.087 |
| Prone positioning during ECMO | 0.75 (0.54 – 1.04) | 0.089 |
| Pneumothorax | 0.50 (0.29 – 0.85) | 0.012 |
| Ventilator associated pneumonia during ECMO | 0.69 (0.49 – 0.98) | 0.038 |
| ECMO duration, per 10 days | 0.96 (0.89 – 1.02) | 0.400 |
| ECMO weaning compliance, per 10mL/cmH_2_O | 1.09 (0.98 – 1.22) | 0.095 |
| Spontaneous breathing | 1.20 (0.81 – 1.78) | 0.360 |

*ECMO* extracorporeal membrane oxygenation*, SOFA* Sequential Organ Failure Assessment, *sHR,* subdistribution hazard ratio
