# Supplementary material for: Mechanical ventilation settings during weaning from venovenous extracorporeal membrane oxygenation
Source: Ann Intensive Care. 2024 Sep 4;14:138. doi: 10.1186/s13613-024-01359-2 (PMC11374948; doi:10.1186/s13613-024-01359-2)
Supplement: Supplementary file 2 — Additional file 2. Association of covariates with the 90-day adjusted probability of successful weaning from mechanical ventilation after ECMO decannulation in the multivariable model analyzing the subgroup of COVID-19 patients, expressed using sHR with their 95% CI. [file 13613_2024_1359_MOESM2_ESM.docx]

**Additional file 2: Association of covariates with the 90-day adjusted probability of successful weaning from mechanical ventilation after ECMO decannulation in the multivariable model in the subgroup of patients with COVID-19, expressed using sHR with their 95% CI**

|  | **Subdistribution hazard ratio.**  **(95% CI)** | ***P* value** |
| --- | --- | --- |
| Age, per 10 years | 0.78 (0.69 – 0.88) | <0.001 |
| Body mass index, per 10 kg/m^2^ | 0.88 (0.74 – 1.04) | 0.140 |
| Pre-ECMO PaO_2_/FiO_2_, per 10mmHg | 0.94 (0.87 – 1.02) | 0.141 |
| Pre-ECMO SOFA | 0.96 (0.93 – 1.00) | 0.047 |
| Renal replacement therapy | 0.78 (0.59 – 1.03) | 0.087 |
| Prone positioning during ECMO | 0.75 (0.54 – 1.04) | 0.089 |
| Pneumothorax | 0.50 (0.29 – 0.85) | 0.012 |
| Ventilator associated pneumonia during ECMO | 0.69 (0.49 – 0.98) | 0.038 |
| ECMO duration, per 10 days | 0.96 (0.89 – 1.02) | 0.400 |
| ECMO weaning compliance, per 10mL/cmH_2_O | 1.09 (0.98 – 1.22) | 0.095 |
| Spontaneous breathing | 1.20 (0.81 – 1.78) | 0.360 |

*ECMO* extracorporeal membrane oxygenation*, SOFA* Sequential Organ Failure Assessment, *sHR,* subdistribution hazard ratio
